# The Epidermis: Redox Governor of Health and Diseases

**DOI:** 10.3390/antiox11010047

**Published:** 2021-12-26

**Authors:** Yosuke Ishitsuka, Dennis R. Roop

**Affiliations:** 1 Department of Dermatology Integrated Medicine, Osaka University Graduate School of Medicine, 2-2 Yamadaoka, Suita, Osaka 565-0871, Japan; 2 Charles C. Gates Center for Regenerative Medicine, Department of Dermatology, University of Colorado Anschutz Medical Campus, Aurora, CO 80045, USA; dennis.roop@cuanschutz.edu

**Keywords:** cornification, genetic diseases, loricrin, KEAP1/NRF2 signaling, epidermal differentiation complex, psoriasis, atopic dermatitis, Langerhans cells

## Abstract

A functional epithelial barrier necessitates protection against dehydration, and ichthyoses are caused by defects in maintaining the permeability barrier in the stratum corneum (SC), the uppermost protective layer composed of dead cells and secretory materials from the living layer stratum granulosum (SG). We have found that loricrin (LOR) is an essential effector of cornification that occurs in the uppermost layer of SG (SG1). LOR promotes the maturation of corneocytes and extracellular adhesion structure through organizing disulfide cross-linkages, albeit being dispensable for the SC permeability barrier. This review takes psoriasis and AD as the prototype of impaired cornification. Despite exhibiting immunological traits that oppose each other, both conditions share the epidermal differentiation complex as a susceptible locus. We also review recent mechanistic insights on skin diseases, focusing on the Kelch-like erythroid cell-derived protein with the cap “n” collar homology-associated protein 1/NFE2-related factor 2 signaling pathway, as they coordinate the epidermis-intrinsic xenobiotic metabolism. Finally, we refine the theoretical framework of thiol-mediated crosstalk between keratinocytes and leukocytes in the epidermis that was put forward earlier.

## 1. Introduction and Overview

### 1.1. What Is Cornification for?

Cornification is an ultimate form of body wall protection [1], in which molecules are packaged (cross-linked), secreted, and then degraded (desquamation). Although keratinization is a shared differentiation program among stratified squamous epithelia that cover wet surfaces (particularly in the hard palate, tongue papilla, or vagina), keratinocytes in the interfollicular epidermis or the follicular infundibulum are exclusively allowed to form the stratum corneum (SC) [2]. Indeed, this imprinting impedes the terminal differentiation of keratinocytes in submerged cultures [3], even if they are freshly isolated from the epidermis. Implantation of epidermal keratinocytes into the immunocompromised host [4] or exposure to the air–liquid interface with the aid of fibrocyte-laden collagen matrix beneath (organotypic culture) [5] allow their differentiation into fully matured phenotypes. With regards to the organotypic setting, however, the anuclear layers that mimic SC continue to pile up without shedding off presumably due to the incomplete development of the secretory system located beneath the SC [5]. Therefore, “true” cornification appears to require constant crosstalk between the epidermal keratinocytes and the surrounding niche (microenvironment) [6].

The differentiation program of epidermal keratinocytes starts following the exit from the proliferative layer (stratum basale) and detachment from the basement membrane [7]. Microenvironmental cues, such as the calcium gradient [8], vitamin D [9], or vitamin A [10,11], profoundly affect cellular fate. The uppermost living layers, stratum granulosum (SG), where the functional cell death program [12,13] takes place, denote anatomical importance. Before being completely flattened and enucleated, the keratinocyte cell membrane is replaced by a specialized structure called cornified cell envelopes (CEs), which is a macromolecular structure composed of protein and lipids [14]. Transglutaminase enzymes catalyze the covalent cross-linkage [ε-(γ-glutamyl) lysine cross-linkage]. The powerful covalent bond is also seen in stabilizing collagen, elastin, or blood clotting [2]. The earlier phase of CE assembly is mandatory for endowing the SC with waterproof property [2,15]. Additionally, transglutaminase 1 [16], CE precursors envoplakin/periplakin/involucrin (IVL) [17], and desmosomal components [18,19,20] appear indispensable. Findings from mouse models correlate with inborn errors in humans [21,22,23], both of which exhibit aberrantly activated immune responses [20,22] secondary to the compromised epidermal barrier. SC’s compromised inside-out/outside-in permeability barrier function, maintained by interstitial lamellated lipids and adhesion molecules [24], has a pathogenic role. It is thus reasonable that the “leaky” SC promotes pathogen sensing of antigen-presenting cells [25] in the uppermost living layer SG, where dendritic cells (DCs) express tight junction (TJ) proteins and sample self [26] or non-self [27] antigens [28,29]. Common loss-of-function (LOF) variants in the filaggrin (*FLG*) gene are an established susceptibility factor for chronic eczema (atopic dermatitis; AD) [30]. The genotype, which is responsible for the common dry skin (ichthyosis vulgaris) [31], decreases the amount of humectant stored in corneocytes and lowers the threshold of local inflammatory responses [32,33]. However, with the contribution of environmental factors [34], *FLG*-deficiency causes AD, ultimately leading to the sequelae of allergic conditions (atopic march) [35,36]. Premature skin peeling (desquamation) may irritate the skin more directly. Desmosomal defects in the SC, caused either by structural defects or enhanced proteolysis, lead to atopic diatheses, such as multiple food allergies [21,22] or *Staphylococcus aureus* (*S. aureus*) colonization [37]. The compromised barrier function can evoke T_H_17-type adaptive immune responses [38]. Staphylococcal colonization is a typical feature of AD [39], and exfoliative toxins target the key adhesion component desmoglein 1 (DSG1) in the SG [40]. Therefore, the “leaky” SC further aggravates local inflammatory responses despite the “preemptive” adaptive immunity [41], constituting an integral part of AD’s vicious cycle.

### 1.2. Revisiting Epidermal Sulfur Metabolism

**Figure 1:**
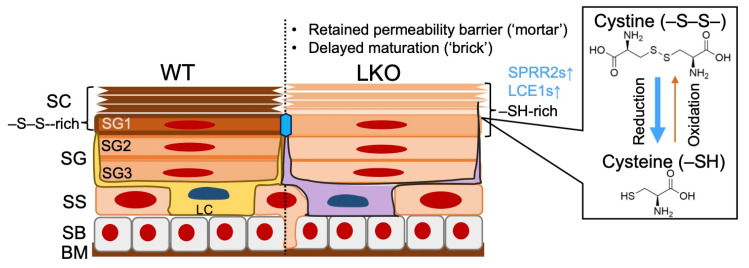
Schematic representation of loricrin-knockout (LKO) epidermis. A thiol (-SH) gradient through the epidermis provides unparalleled cytoprotection, and cornification involves prompt and organized disulfide (–S–S–) cross-linkages. Loricrin is a major effector of cornification, and differentiated layers of LKO epidermis are mechanically compromised despite the presence of other components, such as small proline-rich proteins 2 (SPRR2s) and late cornified cell envelope proteins 1 (LCE1).

The evidence mentioned above highlights the importance of the “mortar” [42] for maintaining skin health and diseases, particularly when focusing on AD pathology [43]. Nonetheless, we need to acknowledge the fundamental principle of cornification, i.e., the metabolism of sulfur; thiol (–SH) groups of the proliferative epidermal layer are converted into strong covalent (–S–S–) bridges of the keratin molecule [44]. Disulfide (–S–S–) bonds reinforce the robustness that protects against a myriad of external insults, such as ultraviolet (UV) rays [45,46,47] or electrophiles [48,49,50]. Not surprisingly, the rudiment is applied to various surface epithelia, such as the squamous mucosa [51], the bronchial epithelium [52,53], or even the gut epithelium [54,55]. Either the excess [55] or the insufficiency [53] of disulfide on the barrier surface leads to the development of pathologies. Loricrin (LOR), which is named after the “lorica”—meaning armor in Latin—[56], is an essential effector of cornification that executes disulfide-mediated cross-linkages [57]. Gene knockout studies have revealed that LOR is nonessential for the “mortar” [15,58] (Figure 1). Nonetheless, the sulfur-rich, major CE constituent protects against harmful stimuli [2]. The findings may epitomize the LOR’s indispensable role as an armor, as the name connotes [56], in terrestrial lifestyles. In a similar vein, the squamous epithelia are equipped with the Kelch-like erythroid cell-derived protein with the cap “n” collar homology-associated protein 1 (KEAP1)/NFE2-related factor 2 (NRF2) signaling pathway [59], which enables prompt xenobiotic responses in the differentiated layers of the squamous epithelium [60]. Dysregulation in the epidermal cellular reduction/oxidation (redox) apparatus can lead to many kinds of skin diseases while being tissue-protective in most situations [60]. In the tissue-protective scenario, we have found that small proline-rich proteins (SPRR2) [57] or late cornified envelope proteins (LCE1) [61] are direct downstream targets of the KEAP1/NRF2 signaling pathway. Given that the epidermal differentiation complex (EDC) is one of the rapidly evolving genetic clusters among terrestrial vertebrates [60,62], the antioxidative backup response appears to help amniotes withstand arid terrestrial environments by promoting tissue repair.

In this review, we take psoriasis and AD, in which cornification is impaired, and epidermal redox imbalance plays a pathological role [48,63]. Intriguingly enough, despite somewhat opposing traits [64,65], they share the susceptible genetic loci EDC that harbors the NRF2-downstream target genes [60]. We also review recent mechanistic insights on cornification by focusing on LOR and the KEAP1/NRF2 signaling pathway, as they coordinate the epidermis-intrinsic xenobiotic metabolism [2,15,60]. Finally, we refine the theoretical framework of thiol-mediated crosstalk between keratinocytes and leukocytes in the epidermis that had been put forward earlier [2,60,66].

## 2. Epidermal Differentiation and Skin Diseases

### 2.1. Psoriasis

Psoriasis is a chronic, immune-mediated skin disease that affects approximately 3.2% of the adult population in the United States regardless of sex differences [67]. Genetic components significantly impact psoriasis pathogenesis. The classic human leukocyte antigen (HLA)-Cw6 allele on the psoriasis-susceptibility 1 (PSORS1), activating variants in the caspase recruitment domain family member 14 (*CARD14*) on the PSORS2, TRAF3 interacting protein 2 (*TRAF31P2*) on the PSORS13, LOF variants in interleukin-36 receptor antagonist protein (*IL36RN*) on the PSORS14, or the protective IL-23 receptor allele may represent the immune-driven nature of psoriatic diseases (Online Mendelian Inheritance in Man (OMIM), accessed on 18 November 2021 [68]). As the remarkable success of immunotherapies targeting the IL-23/IL-17 pathway [69] denotes, inhibiting the rapid polymorphological neutrophil influx that occurs in a noncognate fashion [70] (i.e., the psoriasiform tissue reaction [71]) is likely to be the therapeutic key. Its close association with major histocompatibility class I, such as HLA-Cw6 or HLA-B27 alleles, indicates the autoimmune aspect of this chronic inflammatory condition [72,73]. However, unrestrained innate immune responses (autoinflammation) remain an essential pathological component because nonspecific stimuli, such as trauma, bacterial/viral infection, or drugs, evoke tissue imprinted immunological memory [74]. The quintessential clinical observation is the Köbner phenomenon [75]. Rodent models are vital tools for drug development and experimental pathology. For instance, the HLA-B27 transgenic rat [76], the vascular endothelial growth factor [77]/signal transducers and activators of transcription 3 [78] transgenic mice, the *Il36rn* [79]/*Il1rn* [80] -knockout mice, psoriasis xenograft [81], and topical imiquimod (IMQ) (toll-like receptor 7 agonist) application [82] have revealed important aspects of the pathology of psoriatic diseases. Overall, the success of translational medicine has made psoriasis one of the most successful target diseases in dermatology in recent years [83].

#### 2.1.1. EDC and Psoriasis

In addition to neutrophil infiltration, psoriasis is characterized by hyperplasia and truncated differentiation of the epidermal tissue. Because cellular growth and differentiation are usually exclusive of each other, growth inhibition through blocking nucleic acid synthesis, e.g., methotrexate [67] or epidermal growth factor signaling pathway [84], may rationalize therapeutic efficacy. However, the hyperproliferative psoriatic epidermal phenotype accompanies a distinct immunologically “active” phenotype, in which IL-17-producing, retinoic acid-receptor-related orphan receptor gamma (RORγ)-positive skin resident memory T-cells are enriched [85,86]. Although orally [87] or topically [88] administered vitamin D3 analogs primarily affect the immune system, the fundamental effects on keratinocyte behavior, i.e., inhibiting proliferation [89] and promoting differentiation [9,90], reflect the reversal of the diseased epidermal phenotype. Albeit psoriasis and AD exhibit defective cornification [65], despite their opposing immunological traits, they share risk loci in proximity within the EDC: PSORS4 for psoriasis and ATOD2 for AD [64].

The EDC gene cluster spans 1.6 Mb of the 1q21 human chromosome (mouse 3q) [91], and comprises three gene clusters that encode the following: (i) CE precursor proteins IVL, LOR, SPRRs, and the LCE; (ii) S100 calcium-binding proteins containing EF-hand domains; (iii) and the “fused gene” proteins (S100 fused type proteins), such as *FLG*, hornerin, and repetin that evolved from (i) and (ii) [60]. Given that CE precursor genes, such as the β-globin genes, are supposed to have evolved from common ancestors [92], the host’s adaptive responses on the body surface may have resulted in gene duplication/deletion, ultimately shaping pathologies, such as in sickle cell anemia for the β-globin gene cluster [60]. The *LCE3B*-*LCE3C* deletion represents a psoriasis risk allele in the EDC [93]; however, no other genes, including LOR [94] and *FLG* [95], have been proven as susceptibility genes. Thus, the *LCE3B*-*LCE3C* deletion allele should have some selection advantages to evolve as a psoriasis-susceptibility gene [96].

#### 2.1.2. Antimicrobial Properties of the “Alternative” CE Precursors LCEs

The supposed common origin of the CE precursor genes [92] and the apparently normal-looking phenotype [58] of LOR-knockout (LKO) mice prompted us to investigate other candidates that should be replenished to compensate for the absence of the major CE protein LOR [61]. CE amino acid composition analysis suggested that the candidate should be rich in glycine and serine [97], as well as harbor potential transglutaminase cross-linking sites both in the carboxy and the amino termini [61,92]. This reasoning led us to identify LCE1s, which are located within the LCE gene cluster, as NRF2-downstream target genes [61], as with SPRR2s [57]. These “alternative” CE precursors or “LOR-mimicry” have a generally small molecular weight (approximately 10 kDa) and are robustly induced in response to external stress, such as UVB irradiation, wound healing, and tape-stripping [60]. It is thus reasonable to assume that SPRRs or LCEs are instrumental in protecting against skin surface injuries, and the LKO phenotype may reflect such an adaptive response in the epidermis [15]. In humans, likewise, it appears that LCE3s are particularly sensitive to such micro-injuries [93,98], albeit no significant correlation between the *LCE3B*-*LCE3C* deletion allele and the Köbner phenomenon has been noted [99]. Nonetheless, the LCEs function as antimicrobial peptides [100], as also noted in the other small molecular weight CE precursor SPRRs [101,102]. In this scenario, the *LCE3B*-*LCE3C* deletion results in the transcriptional upregulation of LCE3A, which has exhibited high levels of β-defensin-like broad antimicrobial activity *in vitro* among the LCE3 family [100]. Considering that psoriasis was indeed an exaggerated protective antimicrobial immune response, the upregulated defensin-like activity on the squamous epithelia, including oral mucosa (tonsil/gingiva/pharynx) [60,98], might have protected against invasive streptococcal infections, such as scarlet fever [103]. The inherent molecular property of the LCE that is induced as a result of superficial injuries (and the backup response in LKO mice) [61,93], in combination with the HLA-Cw6 allele-driven immune component, possibly rendered the *LCE3B*-*LCE3C* deletion as the only recurrent psoriasis risk allele within the EDC (PSORS4) [96,104].

### 2.2. Atopic Dermatitis

AD, also called eczema, is the most common inflammatory skin disease that involves early age onset, persistent itch, a relapsing disease course, and which affects 5% to 10% of adults and 10% to 13% of children in the United States [105]. AD reduces the quality of life by compromising sleep quality, work/school associated productivity, interpersonal problems, and self-esteem [105]. AD’s salient immunological trait involves the predominance of type 2 immunity, characterized by the increased risk of local bacterial/viral/fungal infection and systemic IgE-mediated humoral immunity [39]. Epithelium-derived type 2-associated cytokines, such as thymic stromal lymphopoietin (TSLP), IL-25, and IL-33, allow noncognate, local expansion of group 2 innate lymphoid cells, which are a potent producer of IL-13 in the local tissue [106]. Percutaneous exposure of protein antigens promotes B-cell production of high-affinity IgE, which is regarded as the culprit of systemic anaphylaxis [107]. The adaptive immune system plays a pivotal role in this pathogenic compartment; T follicular helper cells producing IL4/IL-13 provide cognate help for B cells to maturate [107].

IL-1α [108] and IL-33 [109] are alarmins that belong to damage-associated molecular patterns that indicate tissue injury contracted by the host [110]. IL-1α possesses strong costimulatory activity and is abundantly expressed in epidermal keratinocytes [108,111]. In the SC of AD patients with LOF *FLG* variants, IL-1α is more abundantly expressed compared to cases without *FLG* mutations [112]. Thus, as the vicious cycle of itch and excoriation (itch-scratch cycle) in AD patients suggests, the epidermal release of danger signals secondary to the defective SC barrier, caused by *FLG*-deficiency [113] or premature skin peeling [114], appears to be the essential element of AD pathology.

Mouse studies have suggested that house dust mites [115], peanut allergens [116], or microbiota [117,118,119], as well as mechanical injuries [119,120,121,122], promote the epidermal release of the alarmins. TSLP signaling constitutes a critical element in initiating allergic inflammation [123]. TSLP acts on DCs [124,125] and allows innate lymphoid cells to expand in the skin [126]. Importantly, TSLP serves as a communication module between epithelial cells and sensory neurons that express transient receptor potential ankyrin 1, thus evoking persistent itch [127]. This evidence makes the TSLP-signaling pathway an attractive therapeutic target in AD [128].

Treatment options for AD had been limited until the emergence of a therapeutic IL-4/IL-13 signaling blockade that utilizes dupilumab, a fully human monoclonal antibody targeting the IL-4 receptor α [129]. Dupilumab improves disease activity by suppressing local/systemic type 2 inflammation and ultimately promoting epidermal differentiation recovery (cornification) [130]. Therefore, dupilumab appears to reverse most of the pathological elements observed in the type 2 inflammation of the skin. However, other emerging therapeutic targets, such as TSLP [128], the “itchy” cytokine IL-31 [131], and Janus kinases [132], will provide a breadth of therapeutic opportunities for AD patients in the future [83].

#### 2.2.1. AD and the FLG Variant

The *FLG* gene encodes a pro-*FLG* protein, which harbors tandemly arranged repeats of *FLG* monomers [113]. During the transition from the SG to the SC, i.e., cornification [1], the huge pro-*FLG* protein undergoes proteolytic cleavage following dephosphorylation (extensively reviewed in [113]). *FLG* monomers are then incorporated CEs, ultimately leading to the generation of natural moisturizing factors that are primarily composed of hygroscopic amino acids in corneocytes [133]. Thus, despite the name connoting the aggregation of intermediate filaments [134], *FLG*’s primary function is to maintain corneocyte humidity. Indeed, inborn errors in the *FLG*-breakdown cascade, as well as LOF *FLG* variants, lead to ichthyotic phenotypes with varied severity and modes of inheritance (OMIM, accessed on 18 November, 2021 [68]).

Ichthyosis vulgaris is the most common inherited disorder of keratinization, and one of the most frequent single-gene disorders in humans [31]. In 2006, Mclean et al. found that the R501X and 2282del4 variants in the *FLG* gene, which lead to the complete absence of gene expression, are a major predisposing factor for AD in European ancestry [30]. Although the prevalence of *FLG* variants shows geographical as well as ethnic differences [135], no other EDC genes [136] have been identified as strong predisposing genes. Although *FLG*-knockout mice have dry, scaly skin and faithfully model the ichthyosis vulgaris phenotype, they do not develop spontaneous AD-like inflammation, even when they are maintained on the BALB/c background [32,137], a high IgE responder. In contrast, the “flaky tail” mice, which harbor spontaneous homozygous frameshift mutation 5303delA in repeat 6 of *FLG*, develop eczema, elevated serum IgE levels [138], and local expansion of group 2 innate lymphoid cells that produce IL-5 when maintained on the BALB/c background [139]. Despite *FLG* genotypes having been reportedly associated with impaired lamellar granule (LG) secretion and decreased levels of tight junction protein expression in humans [140], there might be other confounding factors, apart from those related to the external environment, that affect the interstitial space in the SC [137]. Mouse studies suggest that a nonsense mutation Y208Stop in transmembrane protein 79 (TMEM79), identified in the “flaky tail” mice [141,142], results in spontaneous skin inflammation that is exaggerated in the BALB/c background [139]. Indeed, it has been found that the missense single nucleotide polymorphism in TMEM79 is common among AD patients [141].

TMEM79 is expressed in the trans-Golgi network in the SG, and thus affects the secretory function of LGs [142], which is the prerequisite for SC permeability barrier function [42]. Moreover, TMEM79 regulates mast cell-mediated histaminergic itch, which depends on synergetic effects between keratinocytes and sensory neurons in mice [143]. Moreover, a Xenopus study suggested that the transmembrane TMEM79 protein affects the ectodermal development through the wingless/fizzled signaling pathway, thus revealing a significant impact on the development of integumentary system [144]. Aggregation of evidence suggests the possibility that the “outside-in” hypothesis of AD pathogenesis is not as straightforward as initially anticipated [2].

#### 2.2.2. AD and Corneocyte Adhesion

Cornification begins with the extracellular release of adhesion molecules packaged in LGs to the interstitial space of the SC located apically [2]. Specifically, the homophilic adhesion molecule corneosesmosin (CDSN) plays an essential role. CE replaces keratinocyte cell membrane, and CDSN binds to the outer cell surface, along with long-chain ω-hydroxyceramides that form corneocyte lipid envelopes [2]. After establishing the lipid-rich outercoat, CEs connect CDSN *via* disulfide cross-linkages, and serin protease-mediated proteolysis ensues [145]. Meanwhile, corneocytes keep becoming flattened as they migrate upwards [146] and finally detach from the skin surface [147]. Therefore, successful cornification involves well-coordinated skin peeling (desquamation), which occurs at the very end of the epidermal differentiation [15].

The molecular genetics on inborn errors of skin peeling provides a profound insight into the pathogenesis of AD [148]. Netherton syndrome (NS) is an autosomal recessive trait characterized by ichthyosiform erythroderma, hair shaft abnormality, and atopic manifestations. The patients are born with erythroderma that develops into ichthyosis linearis circumflexia. In addition to eczematous skin lesions, systemic complications are also common, such as failure to thrive (FTT), systemic infection, eosinophilia, increased serum IgE levels, and food allergy [148]. NS is caused by LOF mutations in the serine protease inhibitor of Kasal-type 5 (*Spink5*) gene that encodes lymphoepithelial Kazal-type related inhibitor type 5 (LEKTI). Unopposed proteolytic activity of serine protease kallikreins (KLKs) leads to the degradation of the extracellular domain of DSG1 and CDSN, thus upregulating TSLP in the epidermis [148]. Although *Spink5*-knockout mice exhibit SC detachment and postnatal lethality from dehydration [149], epidermal KLK5 transgenic mice faithfully replicate major symptoms of NS [150]. LOF mutations in the CDSN gene cause autosomal recessive genodermatosis peeling skin syndrome 1 (PSS1), whose symptoms involve ichthyosiform erythroderma, FTT, increased IgE levels, eosinophilia, asthma, urticaria/angioedema, and food allergy [148]. Currently, two independent gene knockout studies of CDSN have been reported [151,152], both of which result in postnatal lethality that phenocopies the *Spink5*-knockout mice [149]. LOF mutations in the *DSG1* gene promote loss of membrane expression of DSG1, thus leading to skin dermatitis, multiple severe allergies, and metabolic wasting (SAM) syndrome [22,148], whose clinical features comprise striate palmoplantar keratoderma, as well as FTT and atopic manifestations similar to NS or PSS1. DSG1-knockout mice exhibit postnatal lethality due to a superficial detachment of the epidermal tissue [19,20], albeit exhibiting T_H_17-associated inflammatory signatures at embryonic day 18.5 (right before birth) [20]. Thus, results from the rodent models and SAM syndrome patients may correspond to immunological alertness (preemptive immunity [41]) augmented by the breach of superficial epidermal tissue [153].

#### 2.2.3. AD as an Antioxidative Response in the Epidermis

As mentioned above, the disrupted SC barrier appears to constitute the major pathogenic element in AD that is followed by systemic foreign antigen-specific IgE responses [36]. Although type 2 inflammation dominates AD pathology, T_H_17 (or type 17) inflammation does have pathogenicity in AD [154], particularly at the disease onset [155].

Because we had characterized the role of the KEAP1/NRF2 signaling pathway that compensates for the loss of a major CE protein LOR [57,61], we examined whether NRF2 activation ameliorates psoriasiform tissue reaction evoked by topical IMQ application [82], in which neutrophil influx to the upper epidermis impairs cornification [71]. Indeed, NRF2-knockout mice suffered exaggerated type 17 responses, including increased expression levels of tumor necrosis factor, IL6, IL23, and IL17a [63]. An electrophile dimethyl fumarate (DMF) is an anti-psoriatic drug that has been empirically used in Europe, and which has recently become a Food and Drug Administration (FDA)-approved drug for relapsing-remitting multiple sclerosis (MS) [156], which is also a T_H_17-driven autoimmune disease. Oral DMF administration resulted in protection from ear swelling responses and increased cytokine expression levels, while NRF2-knockout mice responded much less robustly than the wild-type control [63]. The same tissue-protective effects of the KEAP1/NRF2 signaling pathway were observed in the context of tape-stripping-induced recovery responses in LKO [15] or *Spink5*-knockout mice [157]. The repairing response appears to involve NRF2-mediated upregulation of antimicrobial defense [158] or LG secretory function [15]. NRF2 activation can be detrimental, as postnatal lethality of KEAP1-knockout mice [159] or ichthyotic/chloracne-like phenotypes in constitutively active NRF2 transgenic mice [160] suggest. Therefore, we utilized contact hypersensitivity (CHS) allergic responses against the small molecule called hapten. Haptens are usually electrophilic, and their hydrophobic properties determine the outcome of CHS; hydrophobic (lipophilic) haptens cause irritancy to the uppermost epidermal living layer SG during downward penetration, while innoxious haptens do less [161]. NRF2-knockout mice mounted much weak local immediate-type reactions (a model of urticaria/anaphylaxis) [162], and epidermal keratinocyte harbored a relatively small amount of IL-1α that initiates type 2 immune response [48]. Given that the SC causes “sterile” inflammation when implanted intradermally [163], augmentation of NRF2-mediated antioxidative defense appears to initiate the allergic response in the skin [48]. Indeed, high IL-1α expression levels are observed in AD with LOF *FLG* mutations [112], NS [164], or lamellar ichthyosis [165], as well as the “flaky tail” mice [119], in addition to high NRF2 expression levels [48,57,158]. Earlier mouse studies have suggested that both IL-1α [166] and NRF2 [50,167,168] protect against chemical carcinogens. Likewise, the protective function of IgE against cancers has been suggested both epidemiologically [169] and experimentally [170,171,172]. Therefore, antioxidative response occurring at the frontline defense system could inform the immune system regarding cellular dysregulations [171], which can be caused by not only electrophilic chemicals [48,172] but also physical injuries [173], such as UV [47], helminth infection [169], or AD (also called itch that rashes) [48] as well. In summary, it appears that the epidermis converts a myriad of physical threats into biochemical signaling via the KEAP1/NRF2 system, a thiol-mediated sensor-effector apparatus [59].

## 3. Redox Regulators of Skin Health and Diseases

The protective, differentiated layers of the epidermis are sulfur-rich, and the formation of the heavily disulfide cross-linked appendages, such as the SC, claws (nails), feathers, or hair on the skin surface differentiates amniotes from other vertebrates [174]. In contrast, aquatic mammals appear to have lost such armaments required for terrestrial life. A phylogenetic study suggested that type I/type II keratin gene clusters in cetaceans do not contain K1/K10 (that produces the SC), along with other genes for hair, nail, and tongue papillae, as opposed to basal keratins K5/K14/K9 and stress-associated keratins K6/K16/K17 [175]. Evidence suggests that terrestrial life requires sulfur-rich apparatus as a protective shield (against UV rays or xenobiotics). Conversely, aquatic life prioritizes rapid wound healing since there are no physical threats (and dehydration).

### 3.1. LOR, the “Finisher” of Cornification

Through meticulous ultrastructural observation, we found that LOR is a major CE protein accumulated in the inner cell periphery, and NRF2 upregulates LG secretory functions that lead to increased CDSN expression levels [15]. In the absence of LOR, however, apparently normal-looking corneocytes retained desmosomes, which should be degraded as going upward [147]. These results suggest that cell-intrinsic NRF2 activation in LKO mice maintains SC permeability function and upregulates keratinocyte antioxidants SPRR2s/LCE1s [57,61]. The other important finding is that LOR promotes the structural maturation of desmosomes in the SC (corneodesmosomes). Because LKO mice do not exhibit thickened SC, as in the case of the psoriatic or palmoplantar epidermis [176,177], LOR promotes disulfide-mediated cross-linkages of CDSN to the outer surface of corneocyte. This is in line with our previous findings that LKO CE is less efficient in disulfide-mediated cross-linkages of K1/K10 that are anchored to desmosomes [178]. Collectively, LOR is nonessential for maintaining the SC permeability barrier function unless it acquires the nuclear localizing signal [179], but it is indispensable for the structural maturation of corneocytes [45,58,178].

### 3.2. “Structural Imprinting” of the Cutaneous Immune Effector Functions

In addition to the desmosome that connects adjacent keratinocytes via keratin intermediate filament, the TJ barrier is indispensable for maintaining the inside-out permeability barrier of the epidermis. Impaired TJ function caused by LOF mutations in claudin-1 (*CLDN1*) causes ichthyosis and sclerosing cholangitis in humans [180], and CLDN1-knockout mice die postnatally from dehydration [181] and develop AD-like features when the expression is systematically regulated [182]. Cornification, however, takes place in the uppermost layer of the SG (SG1) [13], which faces the air–liquid interface and does not have TJ [1,13]. We have found an altered epidermal differentiation program in LKO mice, which leaves the K1/K10 less cross-linked and desmosomes undegraded in the SC [15]. The intercellular junctional complex comprises TJ, adherens junction (AJ), and desmosome in the order given in an apical-basal direction [183]. The anatomical principle may indicate that LKO mice have delayed maturation of the junctional complex.

**Figure 2:**
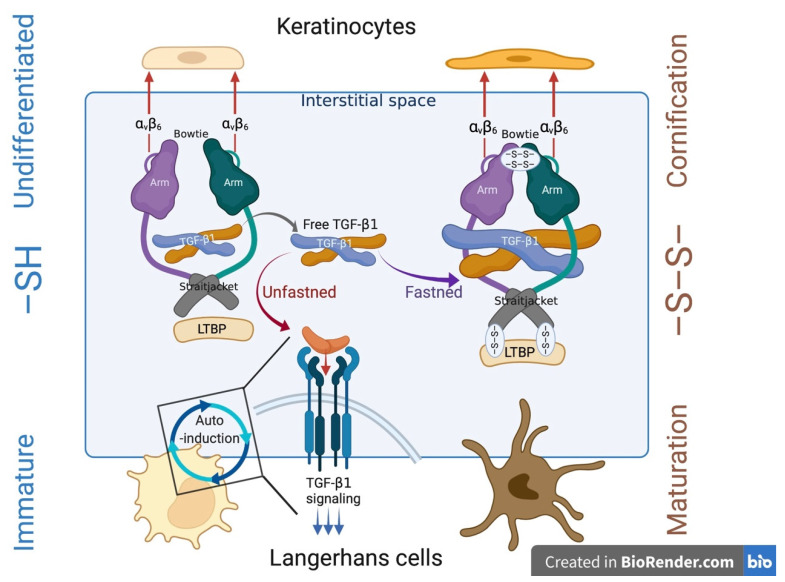
We hypothesized a “Structural imprinting” of the cutaneous immune effector functions. Keratinocytes and Langerhans cells (LCs) might crosstalk via reduction and oxidation [thiol (–SH) and disulfide (–S–S–)] status in the epidermis. Keratinocyte tether LCs via α_v_β_6_ integrin. Epidermal retention of LCs requires the cell-autonomous transform growth factor-beta 1 (TGF-β1) signaling. The prodomain (proTGF-β “fastens” free TGF-β via disulfide cross-linkages in the bowtie and straitjacket regions. The arm domain anchors keratinocytes via α_v_β_6_ integrin and negatively regulates the biological activity of TGF-β in the extracellular matrix. Successful cornification involves disulfide cross-linkages of the junctional component, thus possibly permanently inactivating TGF-β and promoting LC maturation.

Skin DCs survey the interstitial space and capture antigens derived from self [26] or non-self [27] through TJ protein expression on dendrites [28,29]. Interfollicular epidermal keratinocytes anchor Langerhans cells (LCs) through α_V_β_6_ integrin [184], which regulates the activation of transforming growth factor-beta 1 (TGF-β [185]. TGF-β presents in a pro (pro TGF-β1) form in the extracellular matrix, and a free, biologically active TGF-β1 monomer is hidden among the disulfide cross-linked “bowtie” or “straight jacket” domains [185]. Although enhanced antigen priming is a hallmark feature of impaired barrier function, LKO mice exhibited a weak CHS response (unpublished observations). Therefore, we deem that altered epidermal structure caused by the absence of LOR attenuates the immune effector functions (Figure 2). Hence, we could argue that atopy is a unique class of host defense that is allowed to reside in the epidermal tissue. By extension, the structure of the epidermal tissue may be a primary determinant of immune responses that take place on the body surface; LKO mice were resistant in an experimental *S. aureus* colonization model [186]. The “immature” structure of the superficial epidermis may affect the LCs’ behavioral responses evoked by local pathological stimuli [187]. Further investigations are required, and revealing the mechanism may provide us with novel therapeutic interventions that percutaneously control immune-mediated diseases, such as MS [188,189] or food allergy [190,191], instead of vaccinating against plagues [192].

## Abbreviations

AD, ATOD	atopic dermatitis
CARD14	caspase recruitment domain family member 14
CDSN	corneosesmosin
CE	cornified cell envelope
CHS	contact hypersensitivity
CLDN1	claudin-1
DAMP	damage-associated molecular pattern
DC	dendritic cells
DMF	dimethyl fumarate
DSG	desmoglein
EDC	epidermal diffrentiation complex
FLG	filaggrin
FS	failure to thrive
HLA	human leukocyte antigen
IL36RN	interleukin-36 receptor antagonist protein
IMQ	imiquimod
IVL	involucrin
KEAP1	Kelch-like erythroid cell-derived protein with the cap ’n’ collar homology-associated protein 1/NFE2-related factor 2
KLK	kallikrein
LC	Langerhans cell
LCE	late cornified cell envelope protein
LEKTI	lymphoepithelial Kazal-type related inhibitor type 5
LG	lamellar granule
LOF	loss-of-function
LOR	loricrin
MHC	major histocompatibility complex
MS	multiple sclerosis
NRF2	NFE2-related factor 2
NS	Netherton syndrome
OMIM	Online Mendelian Inheritance in Man
PSORS	psoriasis susceptibility
PSS	peeling skin syndrome
redox	reduction and oxidation
ROR-g	retinoic acid-receptor-related orphan receptor gamma
S. aureus	*Staphylococcus aureus*
SC	stratum corneum
SG	stratum granulosum
SPINK5	serine protease inhibitor of Kasal-type 5
SPRR	small prorine-rich protein
TGF-b1	transforming growth factor-beta 1
T_H_	T helper
TMEM79	transmembrane protein 79
TSLP	thymic stromal lymphopoietin
UV	ultraviolet
